# Antifibrinolytic Role of a Bee Venom Serine Protease Inhibitor That Acts as a Plasmin Inhibitor

**DOI:** 10.1371/journal.pone.0032269

**Published:** 2012-02-16

**Authors:** Young Moo Choo, Kwang Sik Lee, Hyung Joo Yoon, Yuling Qiu, Hu Wan, Mi Ri Sohn, Hung Dae Sohn, Byung Rae Jin

**Affiliations:** 1 College of Natural Resources and Life Science, Dong-A University, Busan, Republic of Korea; 2 Department of Agricultural Biology, National Academy of Agricultural Science, Suwon, Republic of Korea; Stanford University, United States of America

## Abstract

Bee venom is a rich source of pharmacologically active substances. In this study, we identified a bumblebee (*Bombus ignitus*) venom Kunitz-type serine protease inhibitor (Bi-KTI) that acts as a plasmin inhibitor. Bi-KTI showed no detectable inhibitory effect on factor Xa, thrombin, or tissue plasminogen activator. In contrast, Bi-KTI strongly inhibited plasmin, indicating that it acts as an antifibrinolytic agent; however, this inhibitory ability was two-fold weaker than that of aprotinin. The fibrin(ogen)olytic activities of *B. ignitus* venom serine protease (Bi-VSP) and plasmin in the presence of Bi-KTI indicate that Bi-KTI targets plasmin more specifically than Bi-VSP. These findings demonstrate a novel mechanism by which bumblebee venom affects the hemostatic system through the antifibrinolytic activity of Bi-KTI and through Bi-VSP-mediated fibrin(ogen)olytic activities, raising interest in Bi-KTI and Bi-VSP as potential clinical agents.

## Introduction

Serine proteases and serine protease inhibitors, which are found in diverse organisms, are of broad interest because they have diverse physiological functions and affect processes, such as the immune response, hemostasis, fibrinolysis, and the elimination of inflammation [1]–[3]. Serine proteases and serine protease inhibitors have been found in snake venom in which many serine proteases exhibit fibrin(ogen)olytic activity [4]–[6] and serine protease inhibitors demonstrate antifibrinolytic activity [7]–[10].

Bumblebee (*Bombus* spp.) venom contains three major components: bombolitin, phospholipase A_2_, and serine proteases [11]–[14]. Our previous studies provided the first evidence of the fibrin(ogen)olytic activity of bumblebee venom serine proteases, which act as prothrombin activators, thrombin-like proteases, and plasmin-like proteases [13], [14]. Although several Kunitz-type serine protease inhibitors have been reported to be present in snake venom [7], [15]–[17], the role of serine protease inhibitors in bee venom remains unknown.

Although bee venom has attracted considerable interest as a rich source of pharmacological substances [18] and has been used traditionally for the treatment of various diseases [19], the mechanism by which bee venom affects the hemostatic system remains poorly understood. In this study, we showed that the bumblebee (*Bombus ignitus*) venom Kunitz-type serine protease inhibitor (Bi-KTI) is a plasmin inhibitor that exhibits antifibrinolytic activity. We also determined how Bi-KTI and *B. ignitus* venom serine protease (Bi-VSP) are involved in fibrinolysis. The present study demonstrates that Bi-KTI acts as an antifibrinolytic agent, providing support for the use of Bi-KTI as a potential clinical agent.

## Results and Discussion

### Bi-KTI is a bee venom Kunitz-type serine protease inhibitor

**Figure 1:**
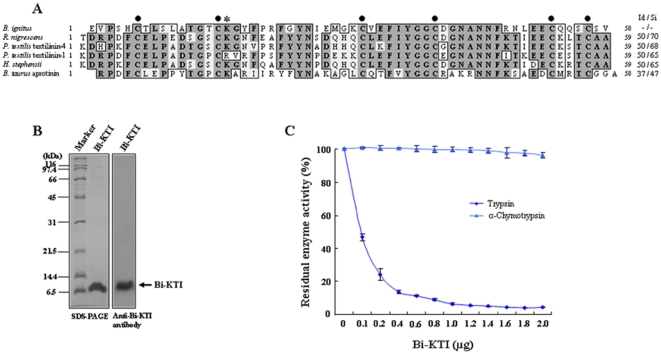
Bi-KTI is a Kunitz-type serine protease inhibitor. (A) The alignment of the amino acid sequences for Bi-KTI and known Kunitz-type serine protease inhibitors. Identical residues are shown in solid boxes. The characteristic cysteine residues are indicated by solid circles. The P1 position is marked with an asterisk. The sources for the aligned sequences were *B. ignitus* (this study, GenBank accession no. JN381496), *Rhinoplocephalus nigrescens* (GenBank accession no. B5KL37), *P. textilis* textilinin-4 (GenBank accession no. Q90W98), *P. textilis* textilinin-1 (GenBank accession no. AF402324), *Hoplocephalus stephensii* (GenBank accession no. B5L5R7), and *Bos taurus* aprotinin (GenBank accession no. P00974). The Bi-KTI sequence was used as a reference for the identity/similarity (Id/Si) values. (B) SDS-PAGE (left) and western blot analysis (right) of purified recombinant Bi-KTI expressed in baculovirus-infected Sf9 insect cells. Recombinant Bi-KTI was identified using an anti-Bi-KTI antibody. (C) Enzyme inhibition by Bi-KTI. Trypsin or chymotrypsin was incubated with increasing amounts of Bi-KTI, and the residual enzyme activity was then determined (*n* = 3).

To explore the role of serine protease inhibitors in bee venom, we identified an expressed sequence tag (EST) for a gene encoding a venom serine protease inhibitor (Bi-KTI) in a *B. ignitus* cDNA library. Bt-KTI consists of 82 amino acids (aa), including a predicted 24-aa signal peptide and a 58-aa mature peptide (GenBank accession number JN381496). Database searches showed that the mature Bt-KTI peptide contains features consistent with snake venom Kunitz-type inhibitors [7], [15]–[17], including six conserved cysteine residues and a P1 site (Figure 1A). Recombinant Bi-KTI was expressed as a 6.5-kDa peptide in baculovirus-infected insect cells (Figure 1B). Using recombinant Bi-KTI, we investigated the inhibitory effects of the enzyme and found that Bi-KTI is a Kunitz-type trypsin-like inhibitor (Figure 1C). Collectively, these data indicate that Bi-KTI is a member of the Kunitz-type inhibitor family [7], [15]–[17].

### Bi-KTI acts as a plasmin inhibitor

**Figure 2:**
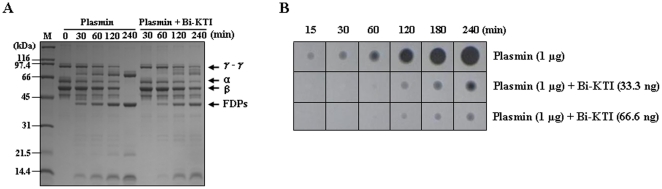
Bi-KTI inhibits plasmin. (A) Bi-KTI-mediated plasmin inhibition assay. The number indicates the time (in min) that fibrin was incubated with plasmin or both plasmin and Bi-KTI. The FDPs are shown. (B) The antifibrinolytic activity of Bi-KTI. Plasmin was dropped onto fibrin plates along with different amounts of Bi-KTI, and the plates were then incubated at 37°C for various periods of time.

Given that Bi-KTI is a Kunitz-type inhibitor [7]–[9], we first assessed whether Bi-KTI inhibits plasmin by determining the time course of human fibrin degradation. We found that Bi-KTI significantly inhibited the degradation of fibrin into fibrin degradation products (FDPs) (Figure 2A). To obtain direct evidence that Bi-KTI inhibits plasmin, we assayed the fibrinolytic activity of this inhibitor on a fibrin plate. Our results showed that the addition of Bi-KTI led to the inhibition of the formation of a clear area (Figure 2B), indicating that Bi-KTI inhibits plasmin by inhibiting the degradation of fibrin into FDPs, which suggests that Bi-KTI has an antifibrinolytic function.

**Figure 3:**
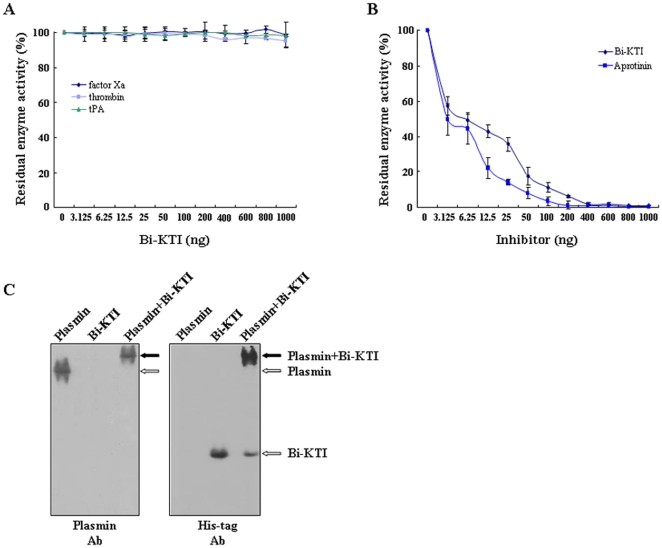
Antifibrinolytic activity of Bi-KTI. (A) Inhibitory activity of Bi-KTI. Factor Xa, thrombin, or tPA was incubated with increasing amounts of Bi-KTI, and the residual enzyme activity was determined (*n* = 3). (B) Comparison of the inhibitory ability of Bi-KTI with that of aprotinin. Plasmin was incubated with increasing amounts of Bi-KTI or aprotinin, and the residual enzyme activity was determined (*n* = 3). (C) Western blot analysis of plasmin-Bi-KTI complex formation via native gel electrophoresis. Three micrograms of plasmin were incubated with 1 µg of Bi-KTI, and the samples (plasmin, Bi-KTI, or plasmin-Bi-KTI mixture) were resolved on a 10% polyacrylamide gel. After electrophoresis, the protein samples were incubated with antiserum against plasmin (left) or His-tag (right). The plasmin, Bi-KTI, or plasmin-Bi-KTI complexes are shown.

We next assayed the ability of Bi-KTI to inhibit important enzymes that belong to the hemostatic system. The results indicate that Bi-KTI has no detectable inhibitory effect on factor Xa, thrombin, or tPA (Figure 3A); however, Bi-KTI strongly inhibited plasmin (Figure 3B), indicating that Bi-KTI has a role as a plasmin inhibitor. We also compared the inhibitory ability of Bi-KTI with that of aprotinin, which is widely used as a plasmin inhibitor [20], [21]. In this experiment, the inhibitory activity of Bi-KTI (IC_50_: 43.53 nM) against plasmin was approximately two-fold weaker than that of aprotinin (IC_50_: 21.66 nM) (Table 1). Similarly, the inhibitory constants (K_i_) of Bi-KTI and aprotinin against plasmin were 3.6 nM and 1.6 nM, respectively (Table 1). To study the mechanism of plasmin inhibition by Bi-KTI, we assessed the formation of plasmin-Bi-KTI complexes via native gel electrophoresis followed by western blotting (Figure 3C). The electrophoretic mobility shift assay showed that Bi-KTI binds to plasmin, indicating the formation of a plasmin-Bi-KTI complex.

**Table 1:** The inhibitory activities of Bi-KTI and aprotinin against plasmin.

	IC_50_ (nM)	K_i_ (nM)
Aprotinin	21.66	1.6
Bi-KTI	43.53	3.6

The antifibrinolytic activity of Bi-KTI due to its ability to inhibit plasmin could help alleviate the bleeding caused by plasmin-mediated fibrin clot digestion [7]–[10], [22]. Consequently, the ability of Bi-KTI to inhibit plasmin suggests that Bi-KTI is an antifibrinolytic agent.

### Antifibrinolytic role of Bi-KTI

**Figure 4:**
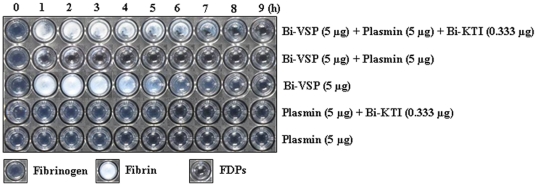
Fibrin(ogen)olytic and antifibrinolytic activities of Bi-VSP and Bi-KTI. The fibrin(ogen)olytic activities of Bi-VSP and plasmin in the presence of Bi-KTI were assayed. Human fibrinogen was incubated with the following: plasmin; plasmin and Bi-KTI; Bi-VSP; Bi-VSP and plasmin; or Bi-VSP, plasmin, and Bi-KTI. The fibrin(ogen)olytic activity was then determined after various periods of time.

Bee venom also contains a Bi-VSP that acts as a fibrin(ogen)olytic serine protease [13]. Thus, we investigated whether Bi-KTI affects Bi-VSP, plasmin, or both during fibrin(ogen)olysis by determining the activity of Bi-VSP and plasmin in the presence of Bi-KTI. When Bi-VSP and plasmin were not treated with Bi-KTI, the fibrinogen was converted into fibrin by Bi-VSP and the fibrin was degraded into FDPs by both Bi-VSP and plasmin (Figure 4). However, the activities of Bi-VSP and plasmin in the presence of Bi-KTI were similar to the activity of Bi-VSP alone (Figure 4). Whereas both Bi-VSP and plasmin were inhibited by Bi-KTI, the activity of Bi-VSP in the presence of plasmin was not significantly affected by Bi-KTI. These results show that Bi-KTI strongly inhibited plasmin during fibrinolysis, indicating that Bi-KTI specifically targets plasmin, as has been demonstrated for textilinin-1, a Kunitz-type inhibitor from *Pseudonaja textilis* venom [7]–[10]. This result further defines a specific role for Bi-KTI as a plasmin inhibitor.

Given that each bee venom component must be in balance with its own function, Bi-VSP and Bi-KTI appear to play important roles in an efficient process because Bi-VSP acts as a fibrin(ogen)olytic agent and Bi-KTI acts as an antifibrinolytic agent. The findings that Bi-VSP activates prothrombin and that Bi-VSP also acts as a fibrin(ogen)olytic protease [13] suggest that Bi-VSP is used to facilitate the spread of bee venom throughout the bloodstream, as has been demonstrated for snake venom fibrin(ogen)olytic enzymes, which remove fibrinogen effectively and, thereby, reduce blood viscosity [23], [24]. Given the similarity in plasmin targeting with textilinin-1, an anti-bleeding agent [7]–[9], Bi-KTI is likely to be an antifibrinolytic agent that reduces bleeding at the sting site of victims. Taken together, our results suggest that prothrombin activation by and the fibrin(ogen)olytic activity of Bi-VSP and the inhibition of plasmin by Bi-KTI may act in a cooperative manner to promote the spread of bee venom under anti-bleeding conditions.

### Conclusion

**Figure 5:**
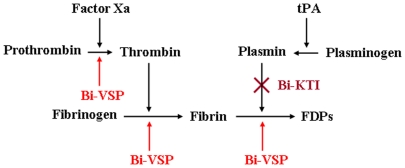
The proposed mechanism for Bi-VSP- and Bi-KTI-mediated fibrin(ogen)olytic and antifibrinolytic activities. Bi-VSP activates prothrombin and degrades fibrinogen into FDPs [13]. Bi-KTI inhibits plasmin, which degrades fibrin into FDPs.

Our results reveal that bumblebee venom affects the hemostatic system through plasmin inhibition by Bi-KTI and through prothrombin activation and Bi-VSP-mediated thrombin- and plasmin-like protease activities (Figure 5). These findings may have therapeutic significance. Although the antifibrinolytic activity of Bi-KTI must be investigated in animal models and human studies, we propose that this protein can function as a clotting factor and may represent a potential clinical agent.

## Materials and Methods

### Gene cloning and sequence analysis

A clone encoding Bi-KTI was selected from the ESTs generated from a cDNA library produced using the venom glands of *B. ignitus* worker bees [13]. Plasmid DNA was extracted using the Wizard Mini-Prep Kit (Promega, Madison, WI) and sequenced using an ABI 310 automated DNA sequencer (Perkin-Elmer Applied Biosystems, Foster City, CA). The sequences were compared using DNASIS and BLAST (http://www.ncbi.nlm.nih.gov/BLAST).

### Protein expression and purification

A baculovirus/Sf9 insect cell expression system [13] was used for the production of recombinant Bi-KTI. A *Bi-KTI* cDNA fragment containing the full-length open reading frame was inserted into the pBAC1 vector (Clontech, Palo Alto, CA) to generate an expression vector that drives the expression of the recombinant protein under the control of the *Autographa californica* nucleopolyhedrovirus (AcNPV) polyhedrin promoter. Recombinant baculoviruses were propagated in Sf9 cells cultured in TC100 medium (Gibco BRL, Gaithersburg, MD) at 27°C. The recombinant proteins were purified using the MagneHis™ Protein Purification System (Promega, Madison, WI). The protein concentrations were determined using a Bio-Rad Protein Assay Kit. SDS-polyacrylamide gel electrophoresis (SDS-PAGE) and western blot analysis were performed as described previously [13] using an Enhanced Chemiluminescence Western Blotting Analysis System (Amersham Biosciences, Piscataway, NJ).

### Measurement of protease and inhibitor activity

Trypsin (400 ng, Sigma) or α-chymotrypsin (400 ng, Sigma) was incubated in 100 mM Tris-HCl (pH 8.0) containing 20 mM CaCl_2_ and 0.05% Triton X-100 with increasing amounts of Bi-KTI at 37°C for 30 min. The residual enzyme activity was determined at 405 nm or 410 nm using the following substrates: 0.4 mM BApNA (Sigma) for trypsin and 0.4 mM Suc-AAPF-pNA (Sigma) for α-chymotrypsin. Additionally, 400 ng of human plasmin (Sigma), thrombin (Sigma), tissue plasminogen activator (tPA; Sigma), or factor Xa (Novagen) was incubated with increasing amounts of Bi-KTI or aprotinin (Sigma) at 37°C for 30 min in 50 mM Tris-HCl buffer (pH 7.4), and the residual enzyme activity was determined at 405 nm using 0.5 mM of chromogenic substrate (Chromogenix, Mölndal, Sweden): S-2251 for plasmin, S-2238 for thrombin, S-2288 for tPA, and S-2222 for factor Xa.

### Fibrinolytic cleavage assay

Human fibrinogen (200 µg, Sigma) that had been clotted with 1 unit of thrombin in 50 mM Tris-HCl buffer (pH 7.4) containing 5 mM CaCl_2_ was incubated with plasmin (500 ng) or both plasmin and Bi-KTI (16.6 ng) at 37°C. The fibrinolytic cleavage was analyzed using 12% SDS-PAGE (10 µg/lane).

### Fibrin plate assay

The fibrin plate assay was performed with 5 ml of human fibrinogen (0.5%) clotted with three units of thrombin. Plasmin or a mixture of plasmin and Bi-KTI was dropped onto the fibrin plates, and the plates were incubated at 37°C for various periods of time. The fibrinolytic activity was determined by examining the formation of a clear area [13].

### Plasmin inhibitory assay

Human plasmin (25 nM, Sigma) was incubated with increasing amounts of Bi-KTI or aprotinin (Sigma) at 37°C for 30 min in 50 mM Tris-HCl buffer (pH 7.4), and the residual enzyme activity was determined at 405 nm using 100 µM S-2251. The initial reaction rate was determined by calculating the slope of the linear portion of the kinetic curve. The inhibitory effect was expressed as the percent reduction in the initial hydrolysis rate; the reaction rate in the absence of inhibitor was taken as 100%. The inhibitor concentration that decreased the rate of hydrolysis by 50% (IC_50_) was determined. The values of the inhibition constants (K_i_) were calculated using the equation K_i_ = IC_50_/(1+S/K_m_) [25].

### Electrophoretic mobility shift assay

Plasmin (three µg) in 50 mM Tris-HCl buffer (pH 7.4) containing 5 mM CaCl_2_ was mixed with 1 µg of Bi-KTI and incubated at 37°C for 1 h. Samples were resolved on a 10% polyacrylamide gel at 4°C. Following electrophoresis, the proteins were blotted onto a sheet of nitrocellulose transfer membrane (Schleicher & Schuell, Dassel, Germany), and the membrane was blocked by incubation in a 1% bovine serum albumin (BSA) solution and then incubated with an antiserum solution (1∶1,000 v/v) against plasmin or the His-tag at room temperature for 1 h. After washing in TBST (10 mM Tris-HCl [pH 8.0], 100 mM NaCl, and 0.05% [w/v] Tween 20), the membrane was incubated with horseradish peroxidase–conjugated anti-mouse IgG diluted 1∶5,000 (v/v). After repeated washing, the membrane was incubated with ECL detection reagents (Amersham Biosciences) and exposed to X-ray film.

### Fibrin(ogen)olytic assay

The fibrin(ogen)olytic assay was performed using a 96-well plate with 200 µl of human fibrinogen (2%) per well. Each of the following was incubated with fibrinogen in 50 mM Tris-HCl buffer (pH 7.4) containing 5 mM CaCl_2_ at 37°C: plasmin; a mixture of plasmin and Bi-KTI; Bi-VSP; a mixture of Bi-VSP and plasmin; or a mixture of Bi-VSP, plasmin and Bi-KTI. The fibrin clotting and FDPs were observed.
